# Gene Action Governing the Inheritance of Stomatal Conductance in Four Wheat Crosses Under High Temperature Stress Condition

**DOI:** 10.3389/fpls.2021.658443

**Published:** 2021-11-16

**Authors:** Kalasapura Thimmappa Ramya, Amasiddha Bellundagi, Harikrishna, Neha Rai, Neelu Jain, Pradeep Kumar Singh, Ajay Arora, Gyanendra Pratap Singh, Kumble Vinod Prabhu

**Affiliations:** ^1^Division of Genetics, ICAR-Indian Agricultural Research Institute, New Delhi, India; ^2^Division of Plant Physiology, ICAR-Indian Agricultural Research Institute, New Delhi, India

**Keywords:** wheat, stomatal conductance, gene action, temperature stress, growth stage

## Abstract

The knowledge pertaining to gene action and interactions involved in the inheritance of a character in different generations is crucial for determining the breeding strategies in crop improvement program. In the present study, the gene action of stomatal conductance was determined in four wheat populations under high elevated temperatures coupled with late sowing at experimental farm, ICAR-Indian Agricultural Research Institute, New Delhi, India. Steady-state SC-1 leaf porometer was used to record stomatal conductance on adaxial and abaxial leaf surface at late boot (Z 49–50), early milk (Z 73), and late milk (Z 77) growth stages. Evidence for nuclear genetic control of stomatal conductance was strong, with large and repeatable genetic difference observed for parents and progeny across all the four crosses. Mean stomatal conductance for genotypes, GW 322 and GW 366, was consistently low at late boot, early milk, and late milk under timely sown, late sown, and very late sown condition, whereas the converse was true for the high-conducting parents, KAUZ/AA//KAUZ and RAC 875. Additive and additive x additive epistatic effects were large and reasonably consistent at three stages and in all crosses. Detection of epistasis and evidence of transgressive segregation suggested that variation for stomatal conductance was under oligo or polygenic control. Thus, it is conceivable that independent alleles at two or more loci could be pyramided into a single family for increased or decreased stomatal conductance. Additive-based gene action also facilitates with simple selection at early generation to improve stomatal conductance in expected direction. This is the first report on estimates of gene action for stomatal conductance of flag leaf under heat stress condition during reproductive and grain filling stage.

## Introduction

Wheat is the most widely grown cereal in temperate environment; in tropical countries, it is grown as winter crop, and high temperature stress is one of the most disadvantageous factors for the production and productivity of wheat in tropical and subtropical environments (Reynolds et al., 2001; Ramadas et al., 2020). Plant’s growth and development involve series of metabolic activities, which are highly sensitive to temperature. Yield penalties are associated with both chronically high temperatures (mean temperature of the growth cycle being 18–25°C, and maximum day temperatures up to 32°C during grain filling) as well as heat shocks, where temperatures greater than 32°C occur during mid- or late reproductive wheat stages, including grain filling (Wardlaw and Moncur, 1995). According to Nagarajan (2005), heat stress, especially at the terminal stage, is seen as a major reason why India has fallen well short of anticipated production. A substantial wheat area is heat stressed due to delayed planting caused mostly by long-duration paddy varieties in the rice–wheat cropping areas (Joshi et al., 2007).

The major mechanism adopted by the plants to keep themselves cool is by keeping the stomata open. This anatomical adaptation permits gas exchange (water loss, carbon dioxide uptake, oxygen release, or uptake) between atmosphere and interior part of the leaf. This function of gas exchange is called stomatal conductance (Taiz and Zeiger, 2010; Francesconi and Balestra, 2020). When ambient temperature is more than optimum plant temperature, stomatal conductance is enhanced which promotes evaporative cooling of leaves to thereby reduce heat stress (Radin et al., 1994). The differences in the vapor pressure deficit between canopy and atmosphere as well as chemical signals synthesized in dehydrating roots mediate stomatal aperture and therefore water flux to the atmosphere. Consequently, a plant is able to tolerate heat stress to some extent by physical changes (guard cell) within the plant body and frequently by creating signals for changing metabolism (Hasanuzzaman et al., 2013; Siddiqui et al., 2015, 2018).

Positive relationships between grain yield and stomatal conductance (SC) were observed under irrigated environments for cotton, wheat, and other crops (Radin et al., 1994; Reynolds et al., 1994; Morison and Lawlor, 1999). Stomatal conductance has been proposed as a selection tool, when measured on multiple plants in a canopy, and is equally effective as carbon isotope discrimination or canopy temperature (Condon et al., 2004). There is a strong relationship between stomatal conductance and canopy temperature, since stomatal conductance has a direct effect on transpirational cooling (Amani et al., 1996; Fischer et al., 1998). Hence, both traits are affected by many of the same environmental and physiological factors. According to Reynolds et al. (2012), under well-watered conditions, stomatal regulation maintains optimal levels of internal CO_2_ concentration to feed the demand for CO_2_ fixation from the Calvin cycle. However, under soil water deficit, there will be a trade-off with the need to maintain a functional water status of leaves (Cornic, 2000; Lawlor and Cornic, 2002). Besides, decreased transpiration rate, CO_2_ assimilation rate (*A*), stomatal conductance (gs), and net photosynthesis (Pn) in tomato were reported in combined stress of drought and heat (Raja et al., 2020). Therefore, under such conditions differences in the vapor pressure deficit between canopy and atmosphere as well as chemical signals synthesized in dehydrating roots mediate stomatal aperture and therefore water flux to the atmosphere. The closure of stomata may increase leaf temperature depending mainly on the radiation load on the canopy but will result in a better water economy or increased transpiration efficiency (Condon et al., 2004). High stomatal conductance permits leaf cooling through evapotranspiration; this along with higher leaf chlorophyll content and stay green character is associated with heat tolerance (Reynolds et al., 1994). However, such differences cannot be detected in high relative humidity environments because the effect of evaporative cooling of leaves is negligible. Nonetheless, leaves maintain their stomata open to permit the uptake of CO_2_, and differences in the rate of CO_2_ fixation may lead to differences in leaf conductance that can be measured using a porometer (Reynolds et al., 2012). Genotypic variations have been reported for stomatal conductance in wheat (Pinter et al., 1990; Fischer et al., 1998; Rebetzke et al., 2003; Condon et al., 2004; Ramya et al., 2016). Genetic variation is repeatable, indicating SC may be targeted for improving adaptation to specific environments (Rebetzke et al., 2003; Ramya et al., 2016). The heritability of stomatal conductance is reasonably high, with reported values typically in the range of 0.5–0.8 (Rebetzke et al., 2003). In a way, we can select individual plants of segregating generations for cooler canopies in later generation. The handheld porometer provides rapid measurement of leaf stomatal conductance in irrigated trials.

High stomatal conductance appeared to be controlled by a single dominant gene in durum wheat (*T. turgidum* L. var. durum) cross (Clarke, 1997). According to single report available till date, gene action was complex with both additive and non-additive genetic effects for expression of leaf conductance (Rebetzke et al., 2003) in three crosses of *T. aestivum*. According to Cossani and Reynolds (2012), studies on genetic basis of heat adaptive traits are poorly studied. The understanding of the genetic action of physiological trait is important to generate basic knowledge to sustain and improve wheat productivity under variable climatic conditions. The increased genetic determination of the variation in stomatal conductance with increasing selection pressure during the three growth stages indicated stomatal conductance as a indicator of heat stress under some genetic control. This paves a way for studying the genetic control of the stomatal conductance in the contrasting heat tolerant and susceptible cultivars. This knowledge would be of importance to clarify the nature of these traits for stress tolerance, by facilitating the dissection of quantitative traits into simpler components.

Direct genetic modification of stomatal conductance shows it as potential for indirect yield improvement under stress. Till date there is only one report on estimates of gene action for stomatal conductance, studied at penultimate leaves under normal condition (Rebetzke et al., 2003). Indeed, there are no reports on estimates of gene action for stomatal conductance of flag leaf under heat stress condition during reproductive and grain filling stage. Therefore, present investigation was undertaken to study the gene action of stomatal conductance at three growth stages under heat stress condition.

## Materials and Methods

### Population Development

Four genotypes, namely KAUZ/AA//KAUZ, RAC 875, GW 322, and GW 366, were selected as parents. Parents (P_1_ and P_2_) for each cross were chosen based on their contrasting stomatal conductance. Crosses were made during 2012–2013 at ICAR-Indian Agricultural Research Institute (ICAR-IARI), New Delhi, between GW 322 X KAUZ/AA//KAUZ, GW 366 X KAUZ/AA//KAUZ, GW 322 X RAC 875, and GW 366 X RAC 875. During summer 2013, F_1_s of four different crosses along with their parents were grown at summer nursery facility at Regional Station, Dalang Maidan, Directorate of Wheat Research, Lahaul -Spiti, Himachal Pradesh, India. F_1_s were self-pollinated to produce F_2_ seed. The F_1_ plants were also back-crossed to P_1_ and P_2_ to develop back-crossed populations BC_1_P_1_ and BC_1_P_2_, respectively. Resulting generations P_1_, P_2_, F_1_, F_2_, BC_1_P_1_, and BC_1_P_2_ from four different crosses were evaluated separately considering each cross as a single experiment.

### Sowing and Heat Treatment

All the experiments were conducted during *Rabi* 2012–2013 and 2013–2014 at experimental farm, ICAR-Indian Agricultural Research Institute, New Delhi, India. The latitude of the research farm is 28° 38'23”N, longitude 77° 09'27'E and altitude is 228.61m above mean sea level. The meteorological data on atmospheric temperature and relative humidity for the crop season from November 2013 to April 2014 were recorded at automatic weather station. The experiment was laid out in two replicated blocks with six generations. Each cross was sown as a separate experiment under three different sowing dates, *viz.* 15 November (timely sowing; TS), 15 December (late sowing; LS), and 6 January (very late sowing; VLS) during 2013. In all the sowing dates, each block consisted eight rows of 3m in which 20 plants per row were maintained with the spacing of 15cm apart between plants. Each block contained 20 plants of each parent, 20 F_1_, 60 F_2_, 20 BC_1_P_1_, and 20 BC_1_P_2_. Standard cultivation practices prescribed for wheat under irrigated conditions were precisely followed. Irrigation was scheduled for 25–30days of interval for normal date of sowing, while under stress condition, the 15–20days intervals were followed as per the recommended package of practices. All the necessary plant protection measures for pest and diseases such as rust were undertaken.

### Measuring Stomatal Conductance

Stomatal conductance (SC) was measured using steady-state SC-1 leaf porometer (Decagon Devices, Inc., USA) version 9, 2005. Three growth stages, *viz.* late boot (Z 49–50), early milk (Z 73), and late milk (Z 77) stages, were considered for recording stomatal conductance on flag leaf. Measurements during heading and anthesis were avoided to overcome the confounding effects of phenology. The sensor head was calibrated in the experimental plot before taking the measurement as per the company’s protocol. Measurements were made by placing sensor head on upper (adaxial) surface and lower surface (abaxial) at the middle portion of the flag leaf of main tiller. Total SC of leaf was obtained by adding SC recordings of abaxial and adaxial surfaces. All the measurements were made between 09.30 and 14.00h in clear sunny days.

### Statistical Analysis

Each cross and sowing dates were considered as different experiment. Each cross was analyzed separately as a mixed linear model using restricted maximum likelihood iteration procedure (REML) with help of SAS, PROC MIXED programme (SAS Institute Inc., 2009) with sowing date and generations deemed as fixed, and blocks as random effects. Accordingly, the means were estimated for each generation of P_1_, P_2_, F_1_, F_2_, BC_1_P_1_, and BC_1_P_2_ for each cross and *a priori* comparisons between selected generation means were tested using non-orthogonal contrast.

Generation mean analysis was used to estimate gene effects for stomatal conductance measured on each growth stage of three different sowing conditions for each cross. Weighted least-squares regression analyses were used to solve for the midparent (m), additive [a], dominance [d], additive×additive [aa], additive×dominance [ad], and dominance×dominance [dd] genetic effects following the models and assumptions described in Mather and Jinks (1971). A simple additive–dominance genetic model containing only “m,” “a,” and “d” effects was first tested using the joint scaling test described in Rowe and Alexander (1980). Adequacy of the genetic model was assessed using a Chi-square goodness-of-fit statistic determined from deviations from this model. It is statistically significant at *p*=0.05, and a full genetic model including all digenic epistatic effects was then tested. Significance of each individual genetic component was tested using Student’s *t* test. Genetic components with *t* test<0.05 were considered different from zero and significant to the model. Observations on individual lines were used to estimate the phenotypic, additive, and non-additive genetic, and environmental/sampling variance components for use in calculating broad-sense (H) and narrow-sense (h^2^) heritability’s and their standard errors (Ketata et al., 1976). Heritability was classified as suggested by Robinson et al. (1949) into low (0–30%), moderate (30.1–60%), and high (>60%).

## Results

### Date of Measurement and Effect of Atmospheric Temperature

**Figure 1:**
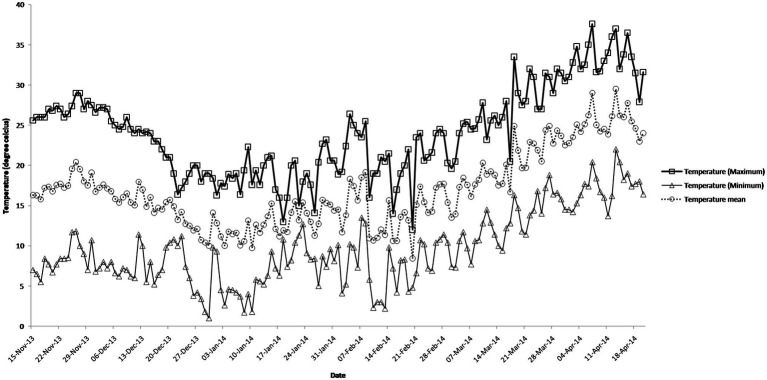
Daily maximum temperature, minimum temperature, and mean temperature in degree Celsius during the crop growth season.

**Figure 2:**
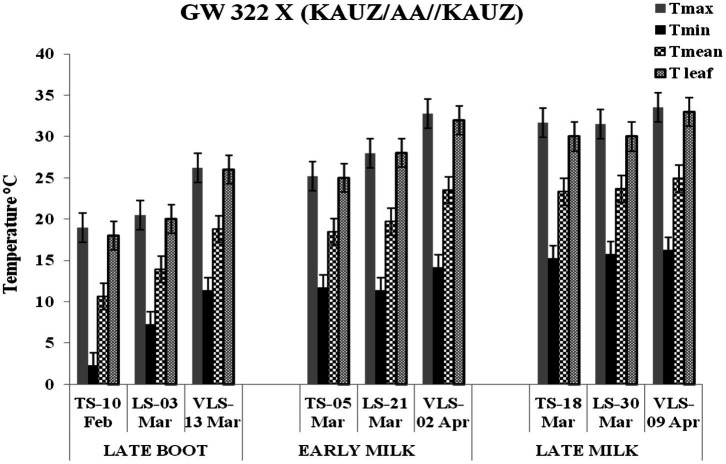
Maximum temperature (Tmax), minimum temperature (Tmin), mean temperature (Tmean), and leaf temperature (Tleaf) in degree Celsius on the date of measuring stomatal conductance at late boot, early milk, and late milk stage under timely sown (TS), late sown (LS), and very late sown (VLS) condition for cross 1—GW 322 X (KAUZ/AA//KAUZ).

**Figure 3:**
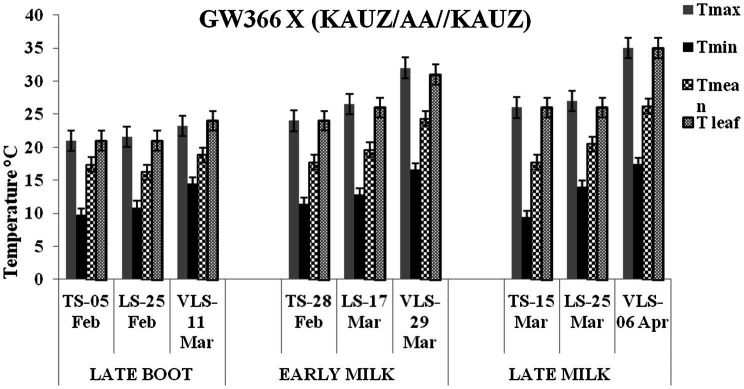
Maximum temperature (Tmax), minimum temperature (Tmin), mean temperature (Tmean), and leaf temperature (Tleaf) in degree Celsius on the date of measuring stomatal conductance at late boot, early milk, and late milk stage under timely sown (TS), late sown (LS), and very late sown (VLS) condition for cross 2—GW 366 X (KAUZ/AA//KAUZ).

**Figure 4:**
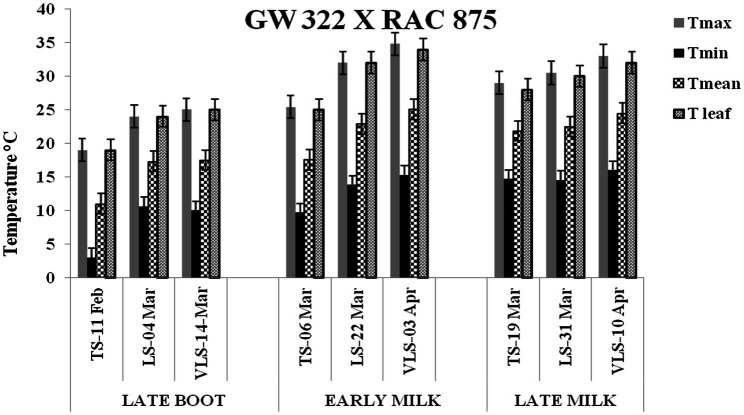
Maximum temperature (Tmax), minimum temperature (Tmin), mean temperature (Tmean), and leaf temperature (Tleaf) in degree Celsius on the date of measuring stomatal conductance at late boot, early milk, and late milk stage under timely sown (TS), late sown (LS), and very late sown (VLS) condition for cross 3—GW 322 X RAC 875.

**Figure 5:**
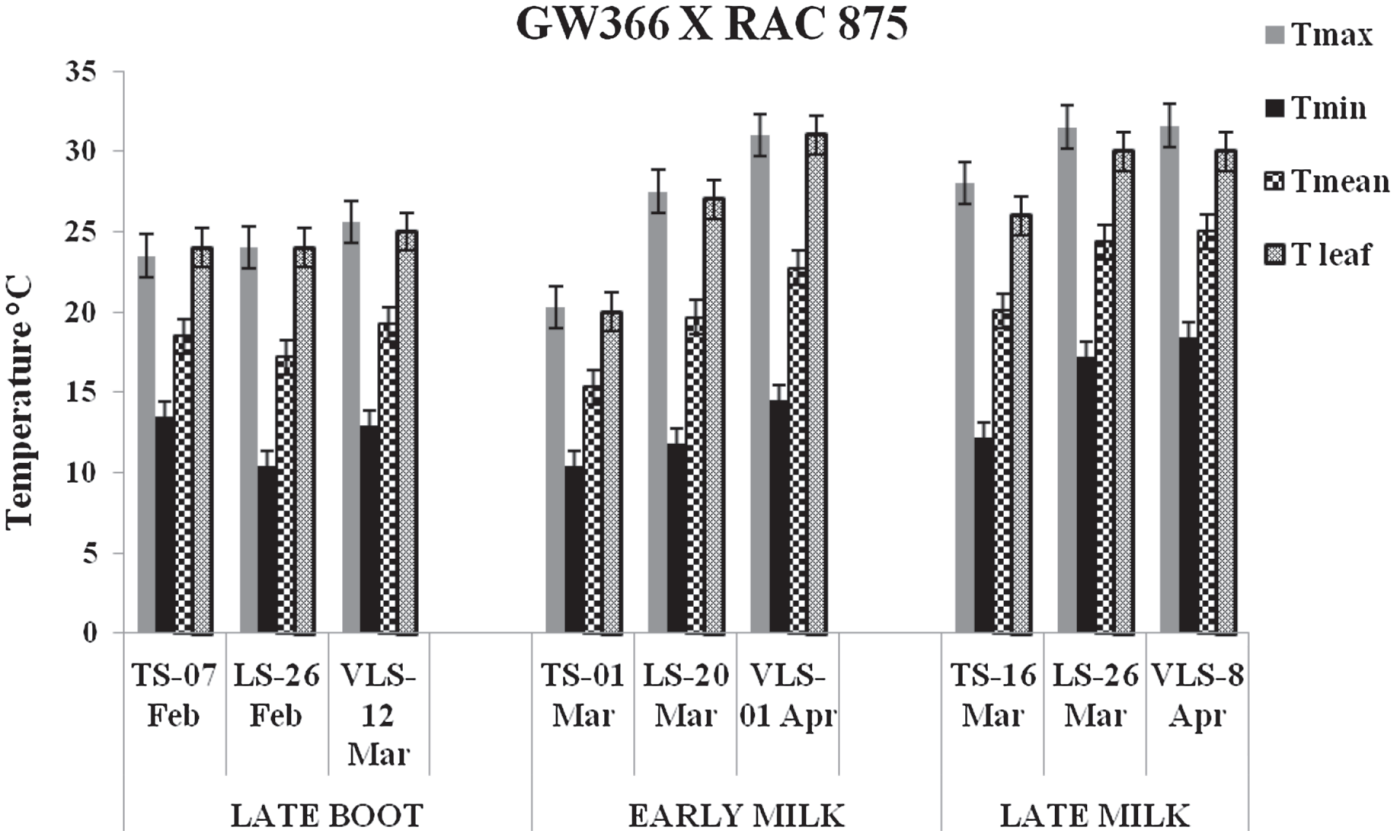
Maximum temperature (Tmax), minimum temperature (Tmin), mean temperature (Tmean), and leaf temperature (Tleaf) in degree Celsius on the date of measuring stomatal conductance at late boot, early milk, and late milk stage under timely sown (TS), late sown (LS), and very late sown (VLS) condition for cross 4—GW 366 X RAC 875.

The daily temperature data indicate heat stress for late and very late sown crop (Figure 1). The maximum, minimum, and mean air temperature along with mean leaf temperature on the date of measurement of stomatal conductance for four crosses at late boot, early milk, and late milk stages under TS, LS, and VLS is given in Figures 2–5. Paired *t* test was used to compare leaf temperatures between sowing dates at three stages. In cross 1 significant differences for mean leaf temperature between TS-LS (*t*=−6.2, *p*<0.01), TS-VLS (*t*=−26.2, *p*<0.01); LS-VLS (*t*=−21.4; *p*<0.01) observed at late boot milk. At early milk, paired *t* test was significant, TS-LS (*t*=−8.2, *p*<0.01), TS-VLS (*t*=−31.1, *p*<0.01); LS-VLS (*t*=−7.3; *p*<0.01). At late boot, paired t test between TS-LS (*t*=−0.25, *p*=0.82), TS-VLS (*t*=−3.3, *p*<0.01); LS-VLS (*t*=−3.0; *p*<0.01). In cross 2 differences in mean leaf temperature between TS-LS (*t*=−0.32, *p*=0.61), TS-VLS (*t*=−3.2, *p*<0.01); LS-VLS (*t*=−3.4; *p*<0.01) at late boot milk. At early milk, paired *t* test was significant, TS-LS (*t*=−5.8, *p*<0.01), TS-VLS (*t*=−31.1, *p*<0.01); LS-VLS (*t*=−27.3; *p*<0.01). At late boot, paired t test between TS-LS (*t*=−0.31, *p*=0.62), TS-VLS (*t*=−33.3, *p*<0.01); LS-VLS (*t*=−29.3; *p*<0.01).

Paired t test was significant for leaf temperature for cross 3 between TS-LS (*t*=−14.8, *p*<0.01), TS-VLS (*t*=−18.1, *p*<0.01); LS-VLS (*t*=−2.3; *p*<0.023) at late boot. At early milk, paired t test was significant, TS-LS (*t*=−19.1, *p*<0.01), TS-VLS (*t*=−21.1, *p*<0.01); LS-VLS (*t*=−5.6; *p*<0.01). At late boot, paired t test between TS-LS (*t*=−5.31, *p*<0.01), TS-VLS (*t*=−9.6, *p*<0.01); LS-VLS (*t*=−5.3; *p*<0.01) was significant. In cross 4 differences in mean leaf temperature between TS-LS (*t*=−0.32, *p*=0.61), TS-VLS (*t*=−2.3, *p*=0.02); LS-VLS (*t*=−2.3, *p*=0.02) at late boot milk was non significant. At late boot, paired t test between TS-LS (*t*=−16.3, *p*<0.01) and TS-VLS (*t*=−16.9, *p*<0.01) was significant; whereas for LS-VLS (*t*=−0.52; *p*=0.21) was non significant.

### Results From Analysis of Variances

There was significant phenotypic variation for stomatal conductance measured in the populations derived from crosses between parents of contrasting stomatal conductance values grown in three different sowing dates. The analyses of variance performed for each cross over sowing date are indicated in Table 1. The magnitude of the main effects and interactions varied between the four crosses. Genotype by sowing date interactions was significant for all the three crosses at all the three growth stages. Mean values for stomatal conductance of parents, F_1_, BC_1_P_1_, BC_1_P_2_, and F_2_ derived groups for each sowing date and for each growth stage are presented in Tables 2 and 3. The contrasts between mean values of six generations were tested by determining Fischer’s least significant different (LSD) at 5% level of probability for significance.

**Table 1:** Mean sum of squares for stomatal conductance during three growth stages in four crosses.

Source	DF-N	DF-D	Cross 1 (GW322 X KAUZ//AA//KAUZ)			Cross 2 (GW366 X KAUZ//AA//KAUZ)			Cross 3 (GW322 X RAC875)			Cross 4 (GW366 X RAC875)		
			Mean square	*F* value	*P*>*F*	Mean square	*F* value	*P*>*F*	Mean square	*F* value	*P*>*F*	Mean square	*F* value	*P*>*F*
Late boot														
Sowing date	2	3	36,275,700	1741.66^**^	<0.0001	18,655,399	392.42^**^	<0.0001	8,563,521	2281.53^**^	<0.0001	25,703,464	16033.2^**^	<0.0001
Generation	5	15	2,258,103	101.77^**^	<0.0001	2,301,397	78.97^**^	<0.0001	2,188,959	46.53^**^	<0.0001	1,418,814	23.61^**^	<0.0001
Sowing date X Generation	10	15	652,343	29.4^**^	<0.0001	397,234	13.63^**^	<0.0001	522,134	11.1^**^	<0.0001	922,210	11.53^**^	0.010
Generation X Block (Sowing date)	15	1,044	22,189	15.4^**^	<0.0001	29,144	6.16^**^	<0.0001	47,040	8.76^**^	<0.0001	60,084	19.06^**^	<0.0001
Error	1,044		1440.948			4733.787			5371.191			3153.053		
Early milk														
Sowing date	2	3	19,991,763	1076.79^**^	<0.0001	3,616,224	121.14^**^	0.0014	5,971,935	162.62^**^	0.0009	3,953,927	395.3^**^	0.0002
Generation	5	15	1,538,729	55.85^**^	<0.0001	1,377,836	74.68^**^	<0.0001	500,024	33.22^**^	<0.0001	650,114	44.81^**^	<0.0001
Sowing date X Generation	10	15	417,828	15.17^**^	<0.0001	94,248	5.11^**^	0.0025	115,848	7.7^**^	0.0003	86,051	5.93^**^	0.0011
Generation X Block (Sowing date)	15	1,044	27,552	20.8^**^	<0.0001	18,449	11.39^**^	<0.0001	15,050	13.35^**^	<0.0001	14,507	20.43^**^	<0.0001
Error	1,044		1324.289			1620.206			1127.761			709.9135		
Late milk														
Sowing date	2	3	23,588,138	360.39^**^	0.0003	17,900,117	1476.7^**^	<0.0001	8,044,536	103.13^**^	0.0017	10,102,155	1174.48^**^	<0.0001
Generation	5	15	822,232	25.74^**^	<0.0001	648,708	20.56^**^	<0.0001	42,345	3.06^**^	0.0424	65,276	8.64^**^	0.0005
Sowing date X Generation	10	15	178,048	5.57^**^	0.0016	91,759	2.91^**^	0.010	139,866	10.1^**^	<0.0001	60,586	8.01^**^	0.0002
Generation X Block (Sowing date)	15	1,044	31,944	19.91^**^	<0.0001	31,551	11.33^**^	<0.0001	13,852	11.05^**^	<0.0001	7559.309	16.17^**^	<0.0001
Error	1,044		1604.692			2784.399			1253.322			467.6287		

DF-N, degrees of freedom numerator; DF-D, degrees of freedom denominator.

** Significant at *p* ≤0.01.

**Table 2:** Mean stomatal conductance mmolm^−2^s^−1^ with their standard error measured on parental, F_1,_ and segregating generations for two crosses GW 322 X (KAUZ/AA//KAUZ) and GW 366 X (KAUZ/AA//KAUZ) under timely, late, and very late sown condition.

		GW 322 X (KAUZ/AA//KAUZ)									GW 366 X (KAUZ/AA//KAUZ)								
		Timely sown			Late sown			Very late sown			Timely sown			Late sown			Very late sown		
Late boot																			
	P_1_	856.1	±	2.21	1052.57	±	4.48	488.69	±	2.82	883.8	±	13.25	1138.47	±	4.52	549.72	±	2.06
	P_2_	1106.1	±	8.05	1586.91	±	3.93	1205.24	±	2.98	1069.44	±	3.44	1585.45	±	4.00	1209.71	±	2.25
	F_1_	911.8	±	10.37	1634.53	±	3.6	799.09	±	2.72	955.47	±	5.05	1621.2	±	3.74	1175.52	±	7.41
	F_2_	915.6	±	4.93	1637.56	±	4.16	794.22	±	4.72	967.14	±	5.61	1610.95	±	6.00	1167.44	±	16.33
	BC_1_P_1_	858.8	±	4.15	1534.26	±	4.61	548.01	±	3.03	873.66	±	6.48	1434.59	±	6.33	1102.67	±	4.01
	BC_1_P_2_	900.0	±	5.26	1712.46	±	6.89	787.24	±	4.96	973.92	±	3.69	1618.98	±	2.81	1288.6	±	4.5
	**LSD(0.05)**	**29.66**			**28.86**			**21.606**			**30.1**			**29.26**			**68.055**		
Early milk																			
	P_1_	756.27	±	1.8	769.29	±	3.51	319.82	±	1.55	586.18	±	4.37	562.79	±	2.97	435.89	±	2.47
	P_2_	890.35	±	8.98	1141.1	±	3.09	833.78	±	1.59	925.05	±	2.19	951.33	±	2.27	831.61	±	2.07
	F_1_	806.28	±	9.21	1188.79	±	4.83	520.96	±	1.65	832.65	±	6.55	969.11	±	2.6	680.79	±	3.53
	F_2_	815.18	±	5.95	1184.43	±	5.83	524.28	±	3.09	823.14	±	5.13	967.85	±	3.63	670.79	±	7.41
	BC_1_P_1_	782.04	±	6.28	1001.11	±	2.86	276.21	±	1.44	761.87	±	7.32	857.67	±	3.9	516.6	±	2.96
	BC_1_P_2_	813.24	±	5.94	1220.14	±	2.16	474.99	±	2.93	815.38	±	5.74	968.19	±	1.84	648.19	±	4.0
	**LSD(0.05)**	**29.68**			**26.07**			**14.17**			**30.16**			**17.62**			**33.475**		
Late milk																			
	P_1_	615.59	±	8.94	475.75	±	2.59	89.49	±	1.06	500.46	±	5.03	478.05	±	3.07	71.72	±	0.91
	P_2_	684.00	±	7.69	933.89	±	2.74	297.92	±	1.06	691.52	±	7.13	933.23	±	2.84	298.52	±	0.93
	F_1_	762.91	±	10.6	729.63	±	2.42	127.13	±	0.79	595.65	±	4.75	692.12	±	3.47	134.09	±	0.89
	F_2_	750.69	±	11.14	734.53	±	3.15	127.91	±	0.86	605.24	±	5.51	697.83	±	9.56	132.2	±	0.7
	BC_1_P_1_	599.86	±	5.55	597.68	±	1.84	93.72	±	0.68	521.5	±	5.97	606.73	±	2.82	127.43	±	0.82
	BC_1_P_2_	753.29	±	7.18	778.2	±	1.51	125.32	±	0.92	529.97	±	6.99	708.41	±	12.49	159.15	±	0.8
	**LSD(0.05)**	**48.23**			**14.69**			**4.06**			**34.91**			**52.72**			**3.77**		

Least significant difference (LSD) at p=0.05 between the generations were provided in bold values.

**Table 3:** Mean stomatal conductance mmolm^−2^s^−1^ with their standard error measured on parental, F_1,_ and segregating generations for two crosses GW 322 X RAC 875 and GW 366 X RAC 875 under timely, late, and very late sown condition.

		GW 322 X RAC 875									GW 366 X RAC 875								
		Timely sown			Late sown			Very late sown			Timely sown			Late sown			Very late sown		
Late boot																			
	P_1_	841.99	±	3.23	1050.24	±	4.87	491.88	±	2.38	852.25	±	2.94	1136.12	±	4.54	547.11	±	2.6
	P_2_	1026.4	±	6.92	1317.13	±	4.78	1120.42	±	4.03	1008.33	±	4.89	1312.66	±	5.13	1116.24	±	2.91
	F_1_	882.39	±	7.2	1389.53	±	7.78	1217.87	±	4.18	1033.68	±	4.61	1509.1	±	3.66	1010.5	±	1.58
	F_2_	878.16	±	6.53	1380.25	±	10.42	1186.04	±	12.66	1048.67	±	7.67	1491.87	±	11.06	1000.3	±	7.99
	BC_1_P_1_	852.73	±	6.4	1150.24	±	8.64	1010.95	±	4.72	921.37	±	3.68	1290.49	±	6.5	770.62	±	4.72
	BC_1_P_2_	929.86	±	8.9	1421.59	±	7.84	1202.95	±	2.94	1,050	±	4.36	1517.62	±	3.6	1160.23	±	3.13
	**LSD(0.05)**	**40.24**			**54.18**			**54.301**			**35.46**			**49.27**			**36.14**		
Early milk																			
	P_1_	787.17	±	8.92	624.01	±	2.81	320.38	±	1.48	588.04	±	4.16	566.55	±	2.45	435.06	±	2.64
	P_2_	863.5	±	3.5	848.74	±	4.02	616.23	±	2.19	860.25	±	2.81	855.06	±	3.17	611.42	±	2.67
	F_1_	762.39	±	6.26	833.16	±	7.76	547.56	±	2.5	791	±	2.91	781.42	±	1.84	531.74	±	1.63
	F_2_	775.97	±	3.9	826.28	±	4.59	541.19	±	5.05	786.93	±	5.32	770.92	±	5.17	532.81	±	1.45
	BC_1_P_1_	778.59	±	3.72	686.11	±	5.17	454.42	±	2.12	635.77	±	2.81	737.73	±	2.16	483.17	±	1.71
	BC_1_P_2_	800.09	±	4.24	847.24	±	4.84	554.93	±	1.5	837.36	±	2.94	778.22	±	2.52	530	±	1.41
	**LSD(0.05)**	**18.46**			**29.09**			**21.905**			**23.19**			**23.47**			**7.176**		
Late milk																			
	P_1_	470.81	±	1.73	379.33	±	2.28	92	±	0.56	479.46	±	1.85	380.63	±	2.22	70.06	±	1.27
	P_2_	572.04	±	9.47	426.44	±	2.58	215.07	±	0.8	589.04	±	8.14	477.24	±	3.15	211.87	±	1.36
	F_1_	563.82	±	8.26	305.86	±	1.74	189	±	0.86	502.04	±	2.48	400.43	±	3.38	86.9	±	1.32
	F_2_	557.14	±	8.46	308.32	±	2.53	187.85	±	4.27	503.11	±	1.92	393.85	±	5.07	86.96	±	0.7
	BC_1_P_1_	472.31	±	5.88	317.16	±	2.28	93.01	±	1.75	484.8	±	3.14	392.52	±	2.3	69.46	±	0.86
	BC_1_P_2_	565.79	±	7.19	354.76	±	2.22	167.9	±	1.4	513.75	±	3.01	334.6	±	2.81	93.72	±	0.62
	**LSD(0.05)**	**36.66**			**11.38**			**18.07**			**14.77**			**22.04**			**3.25**		

Least significant difference (LSD) at p=0.05 between the generations were \provided in bold values.

#### Cross 1—GW322 X (KAUZ/AA//KAUZ)

Genotype, GW 322, was low-conducting parent when compared to KAUZ/AA//KAUZ (Table 2). There was significant contrast between parent 1 (P_1_) and parent 2 (P_2_) at late boot, early milk, and late milk stages of measurement under TS, LS, and VLS. The means of F_1_ and F_2_ generations did not deviate significantly (*p*<0.05). The F_1_ and F_2_ generation means deviated significantly (*p*<0.05) from the mid-parent [MP; where MP=0.5 *(P1+P2)] mean at all the stages and sowing conditions except at early milk under TS. The mean values for BC_1_P_1_ and BC_1_P_2_ deviate significantly (*p*<0.05) at three stages with the back cross means approaching their respective recurrent parent under TS, LS, and VLS. The deviation of means of BC_1_P_1_ and recurrent parent (P_1_) was not statistically significant (*p*>0.05) at late boot, early milk, and late milk stage under TS, the deviations were significant (*p*<0.05) under late and very late sown conditions, and means of BC_1_P_1_ were greater than recurrent parent. However, there was significant deviations (*p*<0.05) between BC_1_P_2_ and recurrent parent at all the stages and sowing dates. The means of BC_1_P_2_ were also significantly (*p*<0.05) lower than the means of recurrent parent at all stages and conditions except at late milk under TS and late boot under LS.

#### Cross 2—GW366 X (KAUZ/AA//KAUZ)

Genotype, GW 366, was low-conducting parent when compared to KAUZ/AA//KAUZ (Table 2). There was significant contrast between parent 1 (P_1_) and parent 2 (P_2_) at late boot, early milk, and late milk stages of measurement under TS, LS, and VLS. The means of F_1_ and F_2_ generations did not deviate significantly (*p*<0.05). The F_1_ and F_2_ generation means deviated significantly (*p*<0.05) from the mid-parent [MP; where MP=0.5 *(P1+P2)] mean at all the stages and sowing conditions except at late boot and late milk (TS) and late milk (LS). The mean values for BC_1_P_1_ and BC_1_P_2_ deviate significantly (*p*<0.05) at three stages with the back cross means approaching their respective recurrent parent under TS, LS, and VLS (except at late milk-LS). The deviation of F_1_ and F_2_ generation means from mid-parent means was non-significant (*p*<0.05) at late boot and late milk stages under TS and at late milk under LS, while deviations were significant (*p*<0.05) at all the stages in VLS. The deviations of BC_1_P_1_ means and recurrent parent means were non-significant at late and late milk under TS, while deviations were statistically significant at rest of the conditions. The means of BC_1_P_1_ were greater than recurrent parent (except at late boot under TS). However, means of BC_1_P_2_ deviated significantly (*p*<0.05) from recurrent parent, while the means of BC_1_P_2_ were lesser than recurrent parent.

#### Cross 3—GW322 X RAC 875

Genotype, GW 322, was low-conducting parent when compared to RAC 875 (Table 3). There was significant contrast between parent 1 (P_1_) and parent 2 (P_2_) at late boot, early milk, and late milk stages of measurement under TS, LS, and VLS. The wsignificant (*p*<0.05). The F_1_ and F_2_ generation means deviated significantly (*p*<0.05) from the mid-parent [MP; where MP=0.5 *(P1+P2)] mean at all the stages and sowing conditions (except for F_2_ at late milk—TS). The deviations of means of BC_1_P_1_ and recurrent parent were non-significant (*p*>0.05) under timely sowing but statistically significant (*p*<0.05) at three growth stages under LS and V LS. The means of backcross were higher than the recurrent parent at all the growth stages under LS and VLS but not at late boot and late milk under TS. The means of BC_1_P_2_ lower than mean of recurrent parent and the deviations were significant at all the stages under TS, LS, and VLS except at early milk under LS condition.

#### Cross 4—GW 366 X RAC 875

Genotype, GW 366, was low-conducting parent when compared to RAC 875 (Table 3). There was significant contrast between parent 1 (P_1_) and parent 2 (P_2_) at late boot, early milk, and late milk stages of measurement under TS, LS, and VLS. The deviations for means of F_1_ and F_2_ generations were not statistically significant (*p*<0.05). The F_1_ and F_2_ generation means deviated significantly (*p*<0.05) from the mid-parent [MP; where MP=0.5 *(P1+P2)] mean at all the stages and sowing conditions. The deviations of means of BC1P1 and recurrent parent were statistically significant (*p*<0.05) at three growth stages under TS, LS, and V LS but non-significant (*p*>0.05), only at late milk under VLS. Indeed, the means of backcross were higher than the recurrent parent except at late milk under TS and VLS. The deviations of means of BC_1_P_2_ and recurrent parent were non-significant (*p*>0.05) for late milk—TS, LS, and significant (*p*<0.05) for rest of the conditions. The means of BC1P2 were lower than means of recurrent parent.

Anderson–Darling test statistic *A*^2^ for all the stages and sowing dates was non-significant (*p*>0.005), indicating goodness of fit for normal distribution of data for F_2_ and BC_1_P_1_. The scaling tests A, B, C, and D deviated significantly from zero since “t” value was significant (*p*<0.01) at three stages under TS, LS, and VLS for all the four crosses studied. In joint scaling test, the Chi-square value was significant (*p*<0.01) for all the crosses and conditions. The lack of fit indicated that simple additive–dominance model was inappropriate and that epistatic gene effects were important in controlling expression of stomatal conductance in progeny of all four crosses under TS, LS, and VLS.

### Goodness of Fit of Genetic Models

The goodness of fit of the seven genetic models for stomatal conductance for each cross at late boot, early milk, and late milk under TS, LS, and VLS sown dates. The selected models represent all possible combinations having the general mean [m], additive [a], dominance [d], and maximum of two interaction components to retain at least one degree of freedom. The goodness-of-fit Chi-square value and probabilities are shown in Tables 4,5. The Chi-square value tested the null hypothesis that the data fit the model, and thus, Chi-square values with *p*≥0.05 denoted goodness of fit. Test for goodness of fit indicated adequacy of more complex models encompassing digenic epistatic effects. The model having [m], [a], [d], [aa], and [ad] genetic components was selected because it had lowest Chi-square value and highest “P” values at all the three stages of measurements under TS, LS, and VLS. Significance of individual genetic components such as general mean [m], additive [a], dominance [d], additive×additive [aa], and additive×dominance [ad] was tested using Student’s *t* test. Genetic components with *t* test<0.05 were considered different from zero and significant to the model.

**Table 4:** Chi-square and probability for goodness-of-fit test of appropriate genetic models for stomatal conductance in two crosses, GW 322 X (KAUZ/AA//KAUZ) and GW 366 X (KAUZ/AA//KAUZ), during three growth stages under timely, late, and very late sown condition.

Stages	Genetic components	CROSS 1—GW 322 X (KAUZ/AA//KAUZ)						CROSS 2—GW 366 X (KAUZ/AA//KAUZ)					
		Timely sown		Late sown		Very late sown		Timely sown		Late sown		Very late sown	
		χ^2^	*P* value	χ^2^	*P* value	χ^2^	*P* value	χ^2^	*P* value	χ^2^	*P* value	χ^2^	*P* value
Late boot													
	m[a]	364.2	<0.001										
	m[a] [aa]									139.17	<0.001		
	m[a][d]	139.16	<0.001	102.63	<0.001	1280.63	<0.001					551.9	<0.001
	m[a][d][aa]	112.72	<0.001	102.3	<0.001	374.15	<0.001	2.92	0.231	28.8	<0.001	536.2	<0.001
	m[a][d][ad]			6.63	0.036							8.66	0.013
	m[a][d][aa][ad]	**0.104**	**0.75**	**0.305**	**0.58**	**0.8**	**0.37**	**2.38**	**0.122**	**2.1**	**0.147**	**0.203**	**0.652**
Early milk													
	m[a]	25.12	<0.001					347.48	<0.001				
	m[a] [aa]			80.36	<0.001					437.1	<0.001		
	m[a][d]	19.75	<0.001									983.4	<0.001
	m[a][d][aa]	14.09	<0.001	60.24	<0.001	285.8	<0.001			317.7	<0.001		
	m[a][d][ad]	5.76	0.056										
	m[a][aa][ad]			30.31	<0.001			14.58	<0.001	1.18	0.55		
	m[a][d][aa][ad]	**0.66**	**0.416**	**0.332**	**0.56**	**0.9**	**0.34**	**1.3**	**0.25**	**0.08**	**0.77**	**1.023**	**0.312**
Late milk													
	m[a]	267.8	<0.001					226.8	<0.001	116.1	<0.001		
	m[ad]	152.5	<0.001										
	m[a] [aa]	193.86	<0.001	680.76	<0.001					113.64	<0.001		
	m[a][d]			125.2	<0.001			75.01	<0.001				
	m[aa][ad]	88.41	<0.001							55.9	<0.001		
	m[a][d][aa]	122.05	<0.001	250.74	<0.001			1.73	0.018	94.4	<0.001		
	m[a][d][ad]											10.67	0.004
	m[d][aa][ad]	34.3	<0.001							24.3	<0.001		
	m[a][d][aa][ad]	**0.31**	**0.57**	**1.52**	**0.21**	**0.45**	**0.5**	**0.85**	**0.355**	**0.31**	**0.57**	**0.5**	**0.48**

Least significant difference (LSD) at p=0.05 between the generations were provided in bold values.

**Table 5:** Chi-square and probability for goodness-of-fit test of appropriate genetic models for stomatal conductance in two crosses, GW 322 X RAC 875 and GW 366 X RAC 875, during three growth stages under timely, late, and very late sown condition.

Stages	Genetic components	CROSS 3—GW 322 X RAC 875						CROSS 4—GW 366 X RAC 875					
		Timely sown		Late sown		Very late sown		Timely sown		Late sown		Very late sown	
		χ^2^	*P* value	χ^2^	*P* value	χ^2^	*P* value	χ^2^	*P* value	χ^2^	*P* value	χ^2^	*P* value
Late boot													
	m[a]												
	m[a] [aa]	1.87	0.6	162.7	<0.001	124.14	<0.001	103.06	<0.001	290.72	<0.001	346.48	<0.001
	m[a][d][aa]	1.8	0.39	129.24	<0.001	122.71	<0.001	33.29	<0.001	290.23	<0.001	309.1	<0.001
	m[a][aa][ad]	**0.31**	**0.856**	44.07	<0.001	**5.06**	**0.08**	21.75	<0.001	52.00	<0.001	**1.6**	**0.45**
	m[a][d][aa][ad]	0.19	0.663	**0.5**	**0.47**	4.65	0.05	**1.73**	**0.188**	**2.18**	**0.14**	1.56	0.21
Early milk													
	m[a]			419.6	<0.001								
	m[a] [aa]	9.13	0.027	107.7	<0.001	433.9	<0.001	350.3	<0.001				
	m[a][d][aa]	8.46	0.014	42.9	<0.001	266.34	<0.001	188.43	<0.001	1014.6	<0.001	202.23	<0.001
	m[a][aa][ad]	**4.36**	**0.113**			107.2	<0.001			9.6	0.008		
	m[a][d][aa][ad]	3.4	0.06	**0.58**	**0.44**	**1.276**	**0.258**	**0.45**	**0.502**	**1.87**	**0.17**	**0.24**	**0.62**
Late milk													
	m[a]	53.2	<0.001					58.05	<0.001				
	m[a] [aa]	50.98	<0.001	23	<0.001								
	m[a][d]							20.45	<0.001	733.01	<0.001		
	m[a][d][aa]	17.1	<0.001	15.73	<0.001	34	<0.001	18.47	<0.001	669.3	<0.001		
	m[a][d][ad]							1.79	0.408				
	m[a][aa][ad]			7.77	0.2					109.65	<0.001		
	m[a][d][aa][ad]	**0.32**	**0.57**	**0.64**	**0.42**	**0.07**	**0.79**	**0.116**	**0.733**	**1.16**	**0.28**	**0.001**	**0.96**

The Chi-square value tested the null hypothesis that the data fit the model, and thus, Chi-square values with *p*>0.05 denoted goodness of fit. Models selected to be best are indicated in bold m=estimated mean; [a]=additive component; [d]=dominance components; [aa]=additive×additive epistatic component; and [ad]=additive×dominance epistatic component. Degrees of freedom for the Chi-square value equal six (total number of generations used to estimate the model) minus the number of components in the model.

#### Cross 1—GW322 X (KAUZ/AA//KAUZ)

Test for goodness of fit indicated adequacy of more complex models encompassing digenic epistatic effects under TS (*p*=0.416 to 0.75), LS (*p*=0.21 to 0.58), and VLS (*p*=0.34 to 0.50) condition (Table 4). At late boot—TS, all the estimates were significant, but epistatic component [ad] was higher than [aa]. Though individual estimate of additive [a] and estimate of dominance [d] were negative indicating influence of high-conducting parent KAUZ/AA//KAUZ, the interaction was positive which reflects the contribution of both the parents to stomatal conductance. However, at early milk stage—TS, individual genetic effect additive [a] was the only significant (*p*<0.05) component and the direction was negative. At late milk—TS, individual genetic effects of additive [a] and dominance [d] was the significant (*p*<0.05) but not the interactions (Table 6) under late sown, all the genetic components were significant (*p*<0.05 for [d] and interaction, *p*<0.01 for [a]) with additive and [aa] epistasis being negative and dominance and [ad] being positive at late boot and late milk. However, [ad] epistasis was negative with small effect at early milk. Under very late sown condition, all the genetic components and interactions were significant (*p*<0.01). Additive and [aa] epistasis were usually negative, while [ad] epistasis was positive at all three stages of measurements.

**Table 6:** Estimates of genetic components, mid-parent [m], additive effect [a], dominance effect [d], additive X additive [aa], dominance X dominance [dd], additive X dominance [ad], standard errors (SE), and Student’s *t* significance level (*P*) for the models with the best Chi-square fit for stomatal conductance measured parents, F_1_, F_2_, BC_1_P_1_ and BC_2_P_2,_ for GW 322 X (KAUZ/AA//KAUZ) and GW 366 X (KAUZ/AA//KAUZ) during three growth stages under timely, late, and very late sown condition.

		CROSS 1—GW 322 X (KAUZ/AA//KAUZ)									CROSS 2—GW 366 X (KAUZ/AA//KAUZ)								
Stages	Genetic components	Timely sown			Late sown			Very late sown			Timely sown			Late sown			Very late sown		
		Estimate	SE	*p* value	Estimate	SE	*p* value	Estimate	SE	*p* value	Estimate	SE	*p* value	Estimate	SE	*p* value	Estimate	SE	*p* value
Late boot																			
	[m]	1122.9^**^	7.33	<0.01	1369.6^**^	11	<0.01	1367.9^**^	13.5	<0.01	1117.13^**^	24.24	<0.01	1728.1^**^	27.6	0.01	793.7^**^	13.3	<0.01
	[a]	−125.04^**^	1.35	<0.01	−267.1^*^	1.64	0.04	−358.27^**^	1.8	<0.01	−96.22^**^	6.09	<0.01	−223.5^**^	4.37	0.01	−330^**^	0.68	<0.01
	[d]	−416.05^*^	12.53	0.019	532.4^*^	20.6	0.02	−1140.2^**^	24.5	0.01	−312.9^*^	42.11	0.017	−219.6ns	49.5	0.14	760.8^**^	21.2	0.01
	[aa]	−141.8^*^	7.2	0.032	−49.8^*^	10.9	0.13	−527^**^	13.37	0.01	−143.5^*^	24.68	0.028	−366.16^*^	27.24	0.04	86.0ns	13.3	0.09
	[ad]	167.63^*^	5.1	0.019	177.9^*^	9.7	0.03	238.09^*^	11.01	0.03	108.2^*^	15.02	0.019	78.2ns	21.9	0.17	288.1^**^	5.6	0.01
Early milk																			
	[m]	883^*^	21.7	0.015	1260.7^**^	9.6	<0.01	1161.18^**^	8.36	<0.01	908.14^*^	28.31	0.019	980.0^**^	3.4	<0.01	1022.2^**^	15.5	<0.01
	[a]	−67.04^*^	3.7	0.035	−185.9^**^	1.3	<0.01	−257^**^	1.05	<0.01	−169.43^**^	2.79	<0.01	−194.2^**^	0.53	<0.01	−197.8^**^	1.6	<0.01
	[d]	−140.8ns	37.9	0.16	−147.4ns	15.5	0.06	−1278.9^**^	15.05	<0.01	−162.77ns	51	0.193	−22.7ns	0.65	0.16	−685.4^*^	25.5	0.023
	[aa]	−59.6ns	21.45	0.22	−305.5^*^	9.5	0.02	−584.3^**^	8.3	<0.01	−152.53ns	28.17	0.116	−223.0^**^	3.4	<0.01	−388.4^*^	15.4	0.025
	[ad]	71.69ns	15.8	0.13	−66.25^*^	4.9	0.04	116.4^*^	6.5	0.035	231.84ns	22	0.06	167.5^**^	2.6	0.01	132.5^*^	8.4	0.04
Late milk																			
	[m]	971.9^**^	28.7	<0.01	878.8^**^	11.4	<0.01	265.5^**^	2.2	<0.01	892^*^	31.28	0.02	846.5^**^	16.1	0.01	147.4^**^	3.4	<0.01
	[a]	−34.2ns	4.68	0.08	−229.0^**^	2.3	<0.01	−104.2^**^	0.5	<0.01	−95.52^*^	5.75	0.04	−227.59^**^	1.1	<0.01	−113.4^**^	0.46	<0.01
	[d]	−429.6ns	47.5	0.07	−294.8^*^	19	0.04	−276.1^**^	3.9	<0.01	−584.5ns	57.32	0.06	−307.4ns	30.9	0.06	−27.1	6.01	0.13
	[aa]	−322.1^*^	28.3	0.05	−174.0^*^	11.5	0.04	−71.8^*^	2.18	0.019	−296.02ns	30.75	0.065	−140.8ns	16.1	0.07	37.6	3.36	0.056
	[ad]	−238.4^*^	17.2	0.04	97.1^*^	7.5	0.04	145.2^**^	1.83	<0.01	174.1ns	26.81	0.09	251.8^*^	14.5	0.03	163.3^**^	2.5	<0.01

Genetic components with *t* test<0.05 were considered different from zero and significant model.

* Significant at *p* ≤0.05;

** Significant at *p* ≤0.01.

#### Cross 2—GW366 X (KAUZ/AA//KAUZ)

Test for goodness of fit indicated adequacy of more complex models encompassing digenic epistatic effects under TS (*p*=0.122 to 0.35), LS (*p*=0.15 to 0.77), and VLS (*p*=0.31 to 0.65) condition (Table 4). Under timely sowing, the individual estimate of additive [a] and estimate of dominance [d] was negative and significant (*p*<0.05 to 0.01) indicating influence of high-conducting parent KAUZ/AA//KAUZ, and the epistasis [ad] was positive which reflects the contribution of both the parents to stomatal conductance. However, at early milk stage—TS, individual genetic effect additive [a] was the only significant (*p*<0.05) component and the direction was negative. At late milk—TS, individual genetic effects, namely additive [a] and dominance [d], were significant (*p*<0.05) but not the interactions (Table 6). Under late sown, additive and its epistasis [aa] genetic components were significant (*p*<0.01) and negative, while [aa] at late milk stage was non-significant (*p*>0.05). Dominance [d] effects were non-significant at all three stages.

Additive x additive epistasis was negative and additive X dominance being positive at all three stages. Under very late sown condition, additive effect was significant (*p*<0.01) negative and [aa] epistasis significant (*p*<0.05) and negative but positive and non-significant at late boot and late milk. Dominance effect was positive and significant (*p*<0.01) at late boot, but negative and significant at early milk and non-significant at late milk. However, additive and dominance epistasis was positive and significant at all the three growth stages.

#### Cross 3—GW 322 X RAC 875

Test for goodness of fit indicated adequacy of more complex models encompassing digenic epistatic effects under TS (*p*=0.11 to 0.85), LS (*p*=0.42 to 0.47), and VLS condition (*p*=0.08 to 0.79; Table 5). However, under timely sown, four models had significantly (*p*=0.39 to 0.85) lower Chi-square value (1.87 to 0.19). The significant genetic components under timely sown were additive and [aa] epistasis being negative in late boot positive at late boot and early milk, while it was negative and non-significant (*p*>0.05) at late milk (Table 7). Under late sown, additive and its epistasis [aa], [ad] genetic components were significant (*p*<0.05) and negative at late boot and early milk, while at late milk stage only [a] and [aa] were positive and significant (*p*<0.05). Under very late sown condition, individual dominant effect was non-significant (*p*>0.05) except at late milk (*p*<0.01), while epistasis [ad] was significant (*p*=0.05 to 0.01) with positive effect at late boot and early milk but with negative effect at late milk. Nevertheless, additive and [aa] epistasis effect was negative and significant (*p*<0.01), and the magnitude of these additive and [aa] epistasis components was also very high in this cross under VLS condition.

**Table 7:** Estimates of genetic components, mid-parent [m], additive effect [a], dominance effect [d], additive X additive [aa], dominance X dominance [dd], additive X dominance [ad], standard errors (SE), and Student’s *t* significance level (*P*) for the models with the best Chi-square fit for stomatal conductance measured parents, F_1_, F_2_, BC_1_P_1_, and BC_2_P_2_ for crosses GW 322 X RAC 875 and GW 366 X RAC 875 during three growth stages under timely, late, and very late sown condition.

Stages	Genetic components	CROSS 3—GW 322 X RAC 875									CROSS 4—GW 366 X RAC 875								
		Timely sown			Late sown			Very late sown			Timely sown			Late sown			Very late sown		
		Estimate	SE	*P* value	Estimate	SE	*P* value	Estimate	SE	*P* value	Estimate	SE	*P* value	Estimate	SE	*P* value	Estimate	SE	*P* value
Late boot																			
	[m]	879.15^**^	1.59	<0.01	1584.8^**^	24.5	<0.01	1211.3^**^	6.85	<0.01	1132.12^**^	13.8	<0.01	1637.7^**^	30.5	0.01	1010.1^**^	1.27	<0.01
	[a]	−92.08^**^	1.5	<0.01	−133.4^**^	2.43	0.01	−314.2^**^	3.7	<0.01	−78.04^*^	3.75	0.03	−88.27^*^	5.06	0.03	−284.5^**^	1.72	<0.01
	[d]	18.46ns	23.12	0.571	−397.2ns	43	0.06	−34.7ns	117.6	0.81	−191.96^**^	56.46	<0.01	−260.7ns	54.6	0.13	−0.66ns	31.03	0.98
	[aa]	54.85^**^	2.29	<0.01	−401.1^*^	44.37	0.03	−405^**^	7.98	<0.01	−201.83ns	31.6	0.09	−413.4^*^	30.1	0.04	−178.77^**^	2.18	<0.01
	[ad]	27.9ns	8.78	0.08	−275.8^*^	17.35	0.04	242.1^**^	35.3	<0.01	−121.17ns	23.7	0.14	−277.7^*^	24.2	0.05	−210.09^**^	10.01	<0.01
Early milk																			
	[m]	774.21^**^	3.8	<0.01	981.9^**^	16.3	0.01	634.8^**^	11.8	0.01	938.12^**^	8.9	<0.01	802.1^**^	12.34	0.01	626.25^**^	3.17	<0.01
	[a]	−37.11^*^	6.89	0.033	−112.3^**^	1.87	0.01	−147.9^**^	1.5	<0.01	−136.1^**^	1.68	<0.01	−144.2^**^	2.74	0.01	−88.18^**^	0.92	<0.01
	[d]	31.08ns	8.13	0.687	−307.7^*^	28.45	0.05	−177.0ns	19.4	0.07	−296.1^*^	15.34	0.03	−42.7ns	21	0.29	−187.8^*^	5.7	0.02
	[aa]	52.55^*^	8.55	0.025	−245.5^*^	16.2	0.04	−166.5^*^	11.7	0.04	−213.9^*^	8.75	0.02	−91.3ns	12	0.08	−103.0^**^	3	0.01
	[ad]	32.26	21.8	0.277	−97.5ns	11.4	0.07	94.8^*^	6.5	0.04	−130.9^*^	6.4	0.03	207.5^*^	8.9	0.02	82.7^*^	2.8	0.02
Late milk																			
	[m]	687.46^**^	17.2	<0.01	285.6^**^	7	0.01	387.5^**^	1.48	<0.01	547.9^**^	3.89	<0.01	568.3^**^	14.6	0.01	162.4^**^	0.13	<0.01
	[a]	50.61*	2.71	0.03	23.5*	1.37	0.03	−61.5^**^	0.13	<0.01	−54.7^**^	1.42	<0.01	48.3^*^	2.1	0.02	−70.9^**^	0.03	<0.01
	[d]	−253.8ns	29.5	0.07	42.03ns	12.6	0.18	−397.1^**^	2.73	<0.01	−90.5^*^	7.2	0.05	−339.8^*^	24.4	0.04	−150.9^**^	0.23	<0.01
	[aa]	−166ns	17	0.06	117.2^*^	6.8	0.03	−234^**^	1.48	<0.01	−13.7ns	3.62	0.16	−139.4ns	14.4	0.06	−21.4^**^	0.12	<0.01
	[ad]	85.75ns	11.8	0.087	28.1ns	5.8	0.13	−26.7^*^	1.2	0.02	−51.69ns	4.11	0.05	−212.4^*^	8.8	0.02	93.2^**^	0.11	0.01

Genetic components with *t* test<0.05 were considered different from zero and significant model.

* Significant at *p*≤0.05;

** Significant at *p*≤0.01.

#### Cross 4—GW 366 X RAC 875

Test for goodness of fit indicated adequacy of more complex models encompassing digenic epistatic effects under TS (*p*=0.18 to 0.73), LS (*p*=0.14 to 0.28), and VLS (*p*=0.21 to 0.96; Table 7). At late boot—TS, individual effects [a] and [d] were negative and significant (*p*<0.05), while epistasis effect [aa] and [ad] was negative and non-significant (*p*>0.05). However, at early and milk, all the genetic components and epistasis effects were negative and significant (*p*<0.05 to 0.01, Table 7). Under late sown, additive and epistasis [aa], [ad] genetic components were negative and significant (*p*<0.05) at late boot stage. At early milk, only [a] and [ad] were significant (*p*<0.05) where epistasis effect was positive. Similarly, at late milk, [a], [d], and epistasis [ad] effects were negative and significant (*p*<0.05). Under very late sown condition, individual dominant effect was non-significant (*p*>0.05) only at late boot and it was negative and significant (*p*<0.01) at early and late milk, while epistasis [aa] and [ad] was significant (*p*=0.05 to 0.01) at all three stages.

### Heritability Components and Narrow Sense Heritability

Variance components such as additive variance (D), dominance variance (H), and environmental variance components and estimated narrow sense heritability for stomatal conductance for four crosses at three growth stages at TS, LS, and VLS are given in Table 8. The heritability ranged from 0.87 to 0.14 under late sown and 0.85 to 0.06 under very late sown situation over the three growth stages. In crosses 1 and 2 where KAUZ/AA//KAUZ was the common parent, environmental variance was high at late boot early milk under TS and at late boot under VLS, so estimating narrow sense heritability was inappropriate. Similar situation existed for cross 3 at early milk—TS, LS, and cross 4 at late milk—TS.

**Table 8:** Estimates of variance components and narrow sense heritability for stomatal conductance in four crosses at three growth stages under timely, late, and very late sown condition.

Growth stages	Timely sown				Late sown				Very late sown			
	Cross 1	Cross 2	Cross 3	Cross 4	Cross 1	Cross 2	Cross 3	Cross 4	Cross 1	Cross 2	Cross 3	Cross 4
Late boot												
E	59.0	71	36.7	17.93	16.16	16.8	35.6	20.1	8.08	21.4	13.1	5.89
D	−41.4	−48.1	−155.06	52.7	−102.8	−23.9	−55.3	134.4	−22.9	460.3	258	63.67
H	−56.5	−61.6	334.02	58.2	210.2	124.6	401.8	140.4	102.6	59.8	71.3	104.7
h^2^ (NS)	–	–	0.72	0.41	0.833	0.20	0.14	0.45	0.26	0.85	0.75	0.36
Early milk												
E	56.2	22.2	43.65	11.21	15.07	6.9	28.08	6.4	2.54	7.6	4.4	5.6
D	−78.5	120.6	−33.25	23.47	42.4	−10.8	−58.26	31.5	−2.18	60.4	37.6	5.63
H	73.85	257.5	−47.17	21.2	−8.9	46.6	88.3	18.1	32.48	68.6	9.2	−2.7
h^2^ (NS)	–	0.76	–	0.42	0.87	0.25	-	0.56	0.06	0.44	0.73	0.66
Late milk												
E	83.8	32.9	53.7	25.3	6.6	9.8	4.95	8.7	0.956	0.83	0.57	1.73
D	83.5	−108.1	−29.3	−30.5	8.5	−145.5	−7.4	25.07	−1.15	−16.53	26.46	−1.26
H	−5.96	205.8	130.4	−25.5	−4.0	616.9	20.75	17.7	1.39	1.93	17.8	−2.4
h^2^ (NS)	0.517	0.83	0.19	-	0.76	0.302	0.41	0.48	-	-	0.59	0.69

E=environmental variance; D=additive variance; H=dominance variance; Cross 1=GW322 X KAUZ//AA//KAUZ; Cross 2=GW366 X KAUZ//AA//KAUZ; Cross 3=GW322 X RAC875; and Cross 4=GW366 X RAC875.

## Discussion

### Effect of Sowing Date on Stomatal Conductance

Atmospheric temperature raised the leaf temperature and stomatal response to elevated temperature at late boot; early milk and late milk stage in four crosses in field conditions were successfully recorded. Staggered sowing done on 15 November (timely sowing, TS), 15 December (late sowing, LS), and 6 January (very late sowing, VLS) was helpful in exposing the plants to different leaf temperatures (Figures 2–5). There were significant differences in temperature at the time of measurement between TS, LS, and VLS and also between the growth stages in each sowing dates. The results were in agreement with the well-established fact that the dependence of leaf temperature on conductance is also *via* transpiration rate (Jones, 1976; Cowan and Farquhar, 1977; Peisker, 1977).

### Gene Action in Four Crosses

Large phenotypic variation was observed for stomatal conductance at all the three growth stages in three sowing dates. Stomatal conductance of the four parents was measured during three crop seasons during 2010, 2011, and 2012 at the experimental site before taking up crossing programme. Genotypes, KAUZ/AA//KAUZ and RAC 875, conducted high during late sown seasons than genotypes GW 322 and GW 366. Differences among individual lines were attributable to various genotypic factors and differential responsiveness of genotypes to variable day temperature. Evidence for nuclear genetic control of stomatal conductance was strong, with large and repeatable genetic differences observed for parents and progeny across all four crosses. Mean stomatal conductance for genotypes, GW 322 and GW 366, was consistently low at late boot, early milk, and late milk under TS, LS, and VLS condition, whereas the converse was true for the high-conducting parents, KAUZ/AA//KAUZ and RAC 875. Nevertheless, there was variation across the stages and sowing dates, but the above-said contrast remains significant. Backcrossing increased the frequency of alleles from the recurrent parent to change the direction of stomatal conductance toward that of the recurrent parent. Stress signals from the roots and consequent production of ethylene resulted in low stomatal conductance in cotton (Sarwar et al., 2018, 2019).

The models described as m[a][d][aa][ad] were consistent and showed the best goodness of fit of the data. However, not all the components in the selected model were significant. Excluding the [m] values, estimates of [aa] and [ad] consistently had the highest significance levels among four crosses at three stages under TS, LS, and VLS. Thus, considering the genetic model goodness of fit and the significance of each of the genetic parameters in the model, [aa] genetic effect was identified as the most important component of the genetic variance for stomatal conductance under normal condition. However, due to elevated temperature under late and very late sowing, [ad] genetic effect was equally important. Additive X additive epistatic effects indicate the need to sample potentially larger populations (Lande and Thompson, 1990). It has been proposed that delaying selection until lines are homozygous should reduce the need for large population sizes (Rebetzke et al., 2006). Both additive and non-additive gene actions were important in controlling expression of stomatal conductance in the four crosses under TS, LS, and VLS. Saint et al. (2010) reported additive x additive genetic effect as single effective component for CT in five crosses when evaluated under rainfed, drought, and heat stress environment. However, dominance and dominance x dominance gene action was also found, though the significance and direction were specific for each environment and genotypic cross. The negative sign on the additive gene effect indicated that additive alleles for lower leaf conductance were largely transmitted for single and interacting loci from low-conducting parents GW 322 and GW 366. There was some evidence for positive [ad] genetic effect in all the four crosses under very late sowing condition which exposed the populations to elevated temperature.

Additive and additive×additive epistatic effects were large and reasonably consistent at three stages and in all crosses (Tables 6 and 7). Evidence for simple additive gene action suggests that replacement and fixation of desirable alleles within a locus could be readily achieved in selection of lines with high or low leaf conductance (Hallauer and Miranda, 1988). However, in the case of epistasis, substitution for desired alleles relies on average effects of specific alleles at other interacting loci. Detection of epistasis and evidence of transgressive segregation suggested that variation for leaf conductance was under oligo or polygenic control. Thus, it is conceivable that independent alleles at two or more loci could be pyramided into a single family for increased or decreased leaf stomatal conductance. Continuous distributions have also been reported for stomatal conductance among segregating tetraploid wheat (*T. dicoccoides* Korn; Carver et al., 1989) and pima cotton progeny (Percy et al., 1996). Stomatal conductance was under additive genetic control in both species. However, there was also strong evidence for epistatic control of stomatal conductance in pima cotton. QTL mapping populations in maize (Sanguineti et al., 1999) and sunflower (Hervé et al., 2001) have identified leaf conductance to be under genetic control at multiple loci. By contrast, Clarke (1997) reported segregation at a single locus for leaf conductance in durum wheat (*Triticum turgidum* L. var. *durum*) cross. There are no reports of maternal gene action for leaf conductance in wheat, and maternal effects influencing photosynthetic capacity may affect CO_2_ movement into the leaf to affect leaf conductance. Rebetzke et al. (2003) reported small and negligible maternal genetic effects.

The complexity in leaf conductance has been suggested to be associated with the fact that different cultivars would have different mechanisms to adapt to particular environmental conditions (Liu et al., 2005). Interactions among genes, stress adaptive traits, and environment would contribute to reduce this additive component. Heritabilities were small and not consistent with the few estimates (Table 8). Clarke (1997) reported a realized heritability estimate of 40% for leaf conductance measured on replicated progeny from a cross between low and high conductance durum wheat. Heritabilities were typically small for leaf conductance when measured on families contained in all replicates. Smaller genetic variance among F_2_ and backcross families reduced heritability estimates (Rebetzke et al., 2003). The absence of a significant [a] effect could then be the result of a complex gene pathway for CT, involving several genes of small effect with different expressions under diverse environments (Mathews et al., 2008). Narrow-sense heritability for bread wheat grown under warm, irrigated conditions was 30% after correction for inbreeding of the parental and progeny generations (Gutierrez-Rodriguez et al., 2000).

Environmental effects on leaf conductance were large. Both growth stages and sowing dates influenced leaf conductance in the four crosses studied. Stomatal aperture is sensitive to a range of environmental factors including temperature, vapor pressure deficit, irradiance, soil–water status, etc. (Farquhar and Sharkey, 1982). Diurnal variation therefore has the potential to confound genotypic differences in leaf conductance. Changes in mean leaf conductance of the different generations throughout each growth stage indicated differential responsiveness and were repeatable in all four crosses. This responsiveness was repeatable in time of day comparisons between low-conducting GW 322 and GW 366 and high-conductive KAUZ/AA//KAUZ and RAC 875. This responsiveness was transmitted to progeny with backcross lines tending toward the recurrent parent as sampling continued later into each stage. Stomatal closure in the afternoon has been observed for older wheat (Fischer et al., 1998) and pima cotton (Lu et al., 1998) varieties grown under irrigated conditions. Similarly, phenotypic correlations for leaf conductance and lint yield in F_2_-derived pima cotton families were significant only when conductance was measured in the afternoon (Radin et al., 1994).

### Implications for Breeding Wheat With Altered Leaf Stomatal Conductance

Many factors can influence a single leaf to affect its stomatal conductance phenotype. Partitioning of this phenotype into its casual genetic components can allow the development of a breeding strategy to enable selection for genes that control leaf stomatal conductance. Maternal genetic effects influencing stomatal conductance were small and not repeatable across all crosses. Thus, selection of the female parent in crossing may not be important. Additive gene effects were moderate in size and were repeatable across sampling stages. Additive-based epistatic effects were also important, indicating the need to sample larger populations in order to recover desired non-allelic combinations. Additive-based gene action also facilitates with simple selection at early generation to improve stomatal conductance in expected direction. The presence of large dominance genetic effects indicates that selection for leaf conductance should be delayed until after some inbreeding when the frequency of heterozygous loci within families has decreased (Hallauer, 1992). The knowledge of additive gene action for stomatal conductance can be used to identify the QTLs/genes governing leaf stomatal conductance.

## Data Availability Statement

The original contributions presented in the study are included in the article/supplementary material, and further inquiries can be directed to the corresponding authors.

## Author Contributions

RK, GS, and KP designed the experiments, and RK performed the statistical analysis. RK, AB, and HK performed the field experiments and collected phenotypic data. NJ, PS, and AA co-wrote the manuscript with RK. GS, NJ, and KP critically revised the manuscript. GS and KP coordinated and supervised the project. All authors contributed to the article and approved the submitted version.

## Conflict of Interest

The authors declare that the research was conducted in the absence of any commercial or financial relationships that could be construed as a potential conflict of interest.

## Publisher’s Note

All claims expressed in this article are solely those of the authors and do not necessarily represent those of their affiliated organizations, or those of the publisher, the editors and the reviewers. Any product that may be evaluated in this article, or claim that may be made by its manufacturer, is not guaranteed or endorsed by the publisher.
